# Social Anxiety in Autistic People: Does the Clark and Wells Model fit?

**DOI:** 10.1007/s10803-023-06108-1

**Published:** 2023-09-26

**Authors:** Alexander C. Wilson, Fiona Gullon-Scott

**Affiliations:** School of Psychology, Newcastle University, Dame Margaret Barbour Building, Wallace Street, Newcastle Upon Tyne, NE2 4DR UK

**Keywords:** Autism, Social anxiety, Cognitive behaviour therapy, Mental health

## Abstract

**Purpose:**

Cognitive behaviour therapy based on the Clark and Wells (1995) model is a first-line treatment for neurotypical people seeking support for social anxiety. While autistic people frequently report high social anxiety, it is unclear how appropriate the model is for this population.

**Methods:**

Over 300 autistic and non-autistic adults completed an online survey measuring key variables of the Clark and Wells model (socially-related negative thoughts, safety behaviours, self-focused attention). Using multiple regression and structural equation modelling, we assessed whether these variables accounted for the link between autism and social fears.

**Results:**

In multiple regression, autistic people experienced greater social fears than expected based on Clark and Wells variables, and safety behaviours were less predictive of social fears in autistic people. In structural equation modelling, Clark and Wells variables only mediated half the link between autistic traits and social fears. In exploratory analysis, we found that distress relating to uncertainty was an additional variable that needed to be taken into consideration in the relationship between autistic traits and social fears.

**Conclusion:**

The Clark and Wells variables were relevant in autism, but did not fully explain elevated social fears in autistic people, which suggests that other factors are also important in accounting for social anxiety in autistic people. This means that therapy informed by the model may not be optimal for autistic people. We recommend further research developing adapted therapy for social anxiety in autistic people.

## Introduction

Social anxiety disorder is diagnosed on the basis of marked fears of social situations where the individual thinks they will be negatively evaluated by others (American Psychiatric Association, 2013). It rarely shows natural recovery (Bruce et al., 2005), and only half of individuals seek treatment, often living with the condition for 15–20 years beforehand (Grant et al., 2005). However, research indicates that social anxiety disorder can be successfully treated through psychological therapy. In a meta-analysis, Mayo-Wilson et al. (2014) found that individual CBT was associated with a large effect size (Hedge’s *g* = 1.19). Guided by this evidence base, the clinical guidelines of the UK National Institute of Health and Care Excellence (NICE, 2019) recommend CBT for social anxiety following one of two models: Clark and Wells (1995) or Rapee & Heimberg (1997). The cognitive-behavioural features of these models are very similar, although the terminology varies, for instance “processing of the self as a social object” in the Clark and Wells model is described in terms of the effects of a “perceived audience” in the other model. For simplicity, we focus on just one model (Clark & Wells, 1995) in this paper, especially as it is associated with the highest treatment effect size in the meta-analysis cited above (*g* = 1.56).

Clark and Wells (1995) proposed that individuals experience negative thoughts about themselves and their social worlds in social situations (e.g., “People will think I am stupid”). Clark and Wells noted that these beliefs often persist even where a person has been exposed to lots of benign social situations where the beliefs might be disconfirmed. Two features are thought to maintain these beliefs: self-focused attention and “safety behaviours”. Focusing only on one’s self and sensations may give a biased picture of the social situation, increasing anxiety and limiting the person’s processing of the actual situation. Safety behaviours are things we do to prevent a feared outcome (e.g. rehearsing our speech if we are afraid of appearing “stupid”), but which leave few opportunities for discovering how significant social threats truly are. In experimental studies, self-focused attention and safety behaviours show causal links with social anxiety (Norton & Abbott, 2016; Piccirillo et al., 2016), and these are the key treatment targets in the social anxiety therapy protocol (Clark et al., 2003). Leigh and Clark (2018) highlight that the Clark and Wells model is unusual in consistently showing superiority to other treatments, including group CBT, exposure therapy, IPT, psychodynamic psychotherapy, fluoxetine, medication-based treatment as usual, and placebo pill.

However, the evidence base has focused on adults in the general population, and it is unknown how well the Clark and Wells model accounts for anxiety in groups whose social skills, challenges and experiences may differ from the general population, such as people on the autism spectrum. Autistic people commonly experience anxiety disorders (Hollocks et al., 2019), and anxiety relating to social situations may be especially common, with estimates as high as half of autistic adults (Spain et al., 2018). The Clark and Wells (1995) model of social anxiety may offer a good basis for understanding and treating social anxiety in autistic people, but it may need to be adapted. While evidence is limited, there is reason to suspect differences in experiences of anxiety relating to social situations in autistic people, as described below.

First, it is important to consider that fears of negative evaluation may have a different reality for autistic people. For a diagnosis of autism, an individual’s social abilities will have been evaluated by a professional authority as different compared to non-autistic people (American Psychiatric Association, 2013). This would seem ripe ground for the development of social anxiety based on an awareness that other people *do* notice social differences about you (although difficulties with perspective-taking in autism may make the opposite true too; Frith, 1989). In addition, autistic people more frequently attract discriminatory, bullying or otherwise negative behaviours from others (e.g., Park et al., 2020) and are more frequently judged negatively at a first impression (Sasson et al., 2017). Autistic people commonly report experiences where they have been made socially vulnerable, for instance through domestic abuse or financial exploitation (Griffiths et al., 2019), and often experience social exclusion, for instance through unemployment even when appropriately qualified (Parsons, 2015). Therefore, autistic people may have different types and/or levels of evidence for the anxiety they may experience in social situations – essentially, their anxiety may be based on considerable negative experience, whereas this may be less characteristic of non-autistic people with social anxiety.

Another important difference might relate to social skills acquisition. Among non-autistic people with social anxiety, avoidance of social situations may reduce opportunities for social learning, and safety behaviours may “contaminate” social situations. However, Clark and Wells (1995) maintain that reduced social skills are not central to social anxiety, and therefore social skills training is not part of therapy. By contrast, autism is characterised by differences in social development, so social skills may play a more causal role in social anxiety in this population, perhaps because individuals have fewer resources for managing social situations. As reviewed by Spain et al. (2018), lower scores in observer-rated social skills relate to social anxiety in autistic people. In addition, when autistic people recognise differences in their social skills compared to their neurotypical peers, they may use learned strategies to mask perceived social difficulties – what has come to be known as “masking” or “camouflaging” (Hull et al., 2019). This may help a person to get by in work/education and reduce risks of being misunderstood, stigmatised or underestimated (Cage & Troxell-Whitman, 2019). However, camouflaging behaviours may also interact with anxiety, possibly with similar negative side effects as safety behaviours (i.e., reinforcing for the person that social catastrophes will happen if they do not perform the behaviour). Pulling these factors together, negative social experiences, social skills and camouflaging may have a specific impact on anxiety in autistic people. This means that the gap between an individual’s socially anxious thoughts and their social reality may be different when comparing autistic and non-autistic people.

In addition, the cognitive-behavioural features of the Clark and Wells model might differ in autism, perhaps due to cognitive differences in autism. South and Rodgers (2017) link alexithymia, discomfort with uncertainty, and atypical sensory function to anxiety in autistic people. These factors, alongside differences in how autistic people might process social information (Frith, 1989), may mean that sensory and cognitive processes in social situations may look different for autistic people. Similarly, some social behaviours may have different functions for autistic people. For instance, avoidance of eye contact may be a safety behaviour for non-autistic people with social anxiety, whereas some autistic people might avoid eye contact due to developmental differences unrelated to anxiety. Therefore, we cannot assume the maintaining factors of social anxiety – self-focused attention and safety behaviours – are equivalent in autistic and non-autistic people.

While anxiety might present in autistic people according to DSM-5 classifications, some anxious presentations in autism may be ‘atypical’ (Kerns et al., 2014). Indeed, relevant to social anxiety, Kerns et al. (2014) found that autistic children sometimes showed social discomfort without fears of negative evaluation (i.e., without the key diagnostic feature of social anxiety in non-autistic groups). Qualitative studies indicate that some autistic adults may experience similar anxiety (Halim et al., 2018; Robertson et al., 2018; Spain et al., 2020). This anxiety may revolve around social uncertainties (e.g., cues, expectations, protocols) rather than fears of negative evaluation per se (Halim et al., 2018). The current study develops on this initial research to examine more comprehensively what social anxiety ‘looks like’ in autism. As it is possible that people may experience distress in social situations without fears of negative evaluation (Kerns et al., 2014), we define social anxiety more broadly than DSM-5, and simply refer to social anxiety as anxiety/distress people experience when together with other people.

The key question of this research is whether the cognitive-behavioural features of social anxiety proposed by Clark and Wells are equally relevant for autistic as well as non-autistic people. This was tested quantitatively using psychometric data collected in a survey. In a pre-registered analysis, we used multiple regression to assess the extent to which the cognitive-behavioural features of the Clark and Wells model account for social fears in autistic people in the same way they do in non-autistic people. We also asked the question in a different way, this time conceptualising autism as a continuum in terms of “autistic traits”. This allowed us to investigate whether a link between autistic traits and social fears could be explained by the cognitive-behavioural features of the Clark and Wells model. In both cases, we hypothesised that the features of the Clark and Wells would not fully account for social fears in autistic people.

## Method

This was a survey-based study that received approval from the Newcastle University Faculty of Medical Sciences Research Ethics Committee (Study No. 2231/13,981). All participants signed a consent form at the start of the survey giving informed consent to participate in the research. A study pre-registration was submitted to the Open Science Framework (OSF) prior to data collection, and data and analysis script are also available on OSF: https://osf.io/bpumn/.

### Participants

We recruited 336 adults aged 18 and over through social media, charities such as Autistica (https://www.autistica.org.uk/), and snowball sampling. Adults were invited to complete a survey if they had internet access, no major uncorrected sensory impairment and no significant history of neurological illness. Four groups were defined for the analysis. Group 1 consisted of 192 adults with a diagnosis of autism made within a clinical service by appropriately trained professionals (such as psychologists, medics and specialist nurse practitioners). Group 2 consisted of 69 “neurotypical” adults. For inclusion in this group, individuals needed to have no neurodevelopmental diagnosis and a score below 6 on the 10-item version of the Autism-Spectrum Quotient (AQ-10; Allison et al., 2012). Group 3 consisted of 51 adults self-identifying as autistic. Group 4 included 24 non-autistic people with an elevated AQ-10 score (6 or over) or neurodevelopmental diagnoses other than autism. Group 4 was retained in the analysis so as not to arbitrarily exclude individuals, and likely reflects some level of neurodivergence/the broad autism phenotype. See Table 1 for demographic characteristics of the sample.

**Table 1:** Background Information about the Sample

Variable	Group 1, Clinical diagnosis of autism (N = 192)	Group 2, Non-autistic group (N = 69)	Group 3, Self-identifying as autistic (N = 51)	Group 4, Broad autism phenotype (N = 24)
Age	40.95 (13.62)	40.30 (17.28)	41.98 (13.95)	32.82 (11.16)
Gender				
* Female*	116 (60)	41 (59)	27 (53)	12 (50)
* Male*	56 (30)	26 (38)	14 (27)	9 (38)
* Non-binary*	20 (10)	2 (3)	9 (18)	3 (13)
* Did not declare*	0 (0)	0 (0)	1 (2)	0 (0)
Race				
* White*	165 (86)	61 (88)	42 (82)	17 (71)
* Black*	0 (0)	4 (6)	1 (2)	1 (4)
* Asian*	3 (2)	1 (1)	2 (4)	3 (13)
* Mixed*	6 (3)	0 (0)	3 (6)	0 (0)
* Did not declare*	18 (9)	3 (4)	3 (6)	3 (13)
Education				
* Less than undergraduate*	44 (23)	17 (25)	13 (25)	7 (29)
* Completed undergraduate degree*	136 (71)	45 (65)	31 (61)	14 (58)
* Current undergraduate*	12 (6)	7 (10)	7 (14)	3 (13)
Neurodevelopmental diagnoses				
* ADHD*	36 (19)	0 (0)	8 (16)	1 (4)
* Dyslexia*	13 (7)	0 (0)	3 (6)	3 (13)
* Dyspraxia/DCD*	15 (8)	0 (0)	0 (0)	1 (4)
* Language/learning-related diagnosis*	9 (5)	0 (0)	1 (2)	0 (0)
Mental health support in the past 12 months				
* Mental health Appointment*	101 (53)	16 (23)	23 (45)	11 (46)
* Brief counselling*	47 (24)	15 (22)	18 (35)	5 (21)
* Course of talk therapy (5 + sessions)*	59 (31)	15 (22)	17 (33)	6 (25)
* Medication*	93 (48)	18 (26)	23 (45)	10 (42)
* Inpatient stay*	9 (5)	0 (0)	1 (2)	0 (0)
Mental health diagnosis				
* Any mental health diagnosis*	159 (83)	33 (48)	33 (65)	14 (58)
* Social anxiety Diagnosis*	58 (30)	12 (17)	15 (29)	5 (21)

Data are Presented as Frequencies (Percentages) Except for Age, which is Given as Mean (Standard Deviation)

We aimed to recruit a sample of at least 150 people, based on power calculation using G*Power. Power was computed for addition of three variables to a simpler multiple regression including four other variables. The smallest meaningful effect size was assumed to be medium-sized (f^2^ = 0.15), which would equate to 7.5% additional variance explained by the three variables over and above 42.5% of variance explained by the other variables. With the alpha level set to 0.05, a sample of 119 is powered at 95% to detect the effect. Allowing for exclusions due to missing data, etc., we aimed to recruit 150 people.

### Measures

The survey took about 20 to 30 min, and began with measures to characterise the sample. These included a bespoke questionnaire recording (1) diagnostic information relating to autism, (2) brief information about mental health history, and (3) demographics (which asked participants via open questions to report their age, gender and race/ethnicity, and gave a set of options to report level of education). There were then several validated self-report questionnaires, as listed below. Then we presented self-report measures of the cognitive-behavioural features of the Clark and Wells model (the SCQ, SBQ and FAQ as described below), as well as some questions eliciting free text responses about experiences of anxiety. All measures have been validated in autistic groups except the measures relating to the Clark and Wells model (the SCQ, SBQ and FAQ). However, it is the aim of this study to highlight the validity (or lack of) of these measures and demonstrate whether they account for social fears in autistic people. Table 2 shows the variables included in the study and the text provides the order of measures given in the survey.

**Table 2:** Variables

Measure	Variable
AQ-10	Autistic traits
GAFS-8	Alexithymia
ASA-A	Anxiety
HADS-D	Depression
CAT-Q	Camouflaging/masking
VEQ-subset	Adverse childhood social experiences
LSAS-SR	Social fears
SCQ	Socially-related negative thoughts
SBQ	Safety behaviours
FAQ-self	Self-focused attention

*Note. AQ-10 = Autism Spectrum Quotient – 10 item version; GAFS-8 = General Alexithymia Factor Score; ASA-A = Anxiety Scale for Autism-Adults; HADS = Hospital Anxiety and Depression Scale; CAT-Q = Camouflaging Autistic Traits-Questionnaire; VEQ = Vulnerability Experiences Quotient; LSAS-SR = Liebowitz Social Anxiety Scale-Self Report; SCQ = Social Cognitions Questionnaire; SBQ = Social Behaviours Questionnaire; FAQ = Focus of Attention Questionnaire*

#### Autism Spectrum Quotient (AQ-10; Allison et al., 2012)

This is a ten-item questionnaire measuring autistic traits, which has been shown to have high sensitivity and specificity for autism. A score of 6 or more indicates possible autism. The AQ-10 is suggested by NICE as a screener for autism in adults (NICE, 2012).

#### General Alexithymia Factor Score (GAFS-8; Williams et al., 2021)

This is a set of eight items taken from the Toronto Alexithymia Scale (TAS-20; Bagby et al., 1994). The questionnaire measures alexithymia, which refers to a difficulty in identifying and describing emotional states. This short version of the TAS-20 shows good psychometric properties and was specifically developed and validated for autistic people.

#### Anxiety Scale for Autism-Adults (ASA-A; Rodgers et al., 2020)

This is a 20-item questionnaire developed and validated to measure anxiety specifically in autistic adults. The validation study demonstrated best model fit for a bifactor model with a strong general anxiety factor accounting for variance across all items, as well as three group factors: uncertainty (5 items), anxious arousal (9 items) and social (6 items). As multidimensionality was not extreme, the validation study supported the use of either a total score across all items or scores on the three subscales, and suggested that scores of 28 and above may indicate clinically elevated anxiety. Note that the social factor equates to social anxiety as traditionally defined (by DSM etc.), as it focuses on anxiety about being observed, evaluated and making a mistake in front of other people. Therefore, it is a somewhat more restricted concept than social fears as explored in this project.

#### Depression Subscale of the Hospital Anxiety and Depression Scale (HADS-D; Zigmond and Snaith, 1983)

This is a 7-item questionnaire measuring low mood/depressive symptoms. The HADS has been found to show the same factor structure in autistic and non-autistic people, and shows robust psychometric properties (Uljarevic et al., 2018). A score of 8 or above suggests clinically significant low mood.

#### Camouflaging Autistic Traits Questionnaire (CAT-Q; Hull et al., 2019)

This is a 25-item questionnaire measuring camouflaging behaviours. These are strategies an individual uses to make their autism less visible in social situations. The measure was validated with autistic people.

#### Subset of Items from the Vulnerability Experiences Quotient (VEQ-subset; Griffiths et al., 2019)

This questionnaire was developed collaboratively with autistic people who determined which negative life experiences are particularly relevant to autistic people. A shortened version was used in this study including only nine items relevant to adverse childhood social experiences (e.g., “As a child, an adult humiliated, embarrassed or scared me.”). In the original version, participants were asked about lifetime experience, with a response format of “yes”, “no”, or “no opportunity”. In this study, the response format was adapted to collect more fine-grained data to a three-point scale: “never”, “once or a small number of times”, “many times”.

#### Liebowitz Social Anxiety Scale – Self Report (LSAS-SR; Liebowitz, 1987)

In this measure, participants rate their level of social fears in 24 situations on a 4-point Likert scale: “none”, “mild”, “moderate”, and “severe”. The measure asks about fear and avoidance, but in the interests of time, we only asked about fear. Fear and avoidance are highly correlated, so it is unlikely we lost information by only asking about fear. The LSAS-SR was used as the key dependent variable in this project, as it purely measures fear and does not ask about beliefs. This is important, as social beliefs may vary across autistic and non-autistic people, and beliefs may show different levels of influence on fear across autistic and non-autistic people. The measure shows promising psychometric qualities in autistic people (Boulton & Guastella, 2020).

#### Social Cognitions Questionnaire (SCQ; Clark, 2005)

This is a 22-item questionnaire measuring negative automatic thoughts that frequently occur in social situations. Participants respond to items (such as “People won’t like me”) on a 5-point scale from “Thought never occurs” to “Thought always occurs when I am nervous”. This questionnaire was devised for use in therapy following the Clark and Wells model of social anxiety as a weekly outcome measure.

#### Social Behaviours Questionnaire (SBQ; Clark, 2005)

This is a 28-item questionnaire measuring safety behaviours people engage in to prevent negative things happening in social situations. Participants respond to items (such as “Make an effort to get your words right”) on a 4-point scale from “Never” to “Always”. Like the SCQ, this measure was devised for therapy following the Clark and Wells model of social anxiety.

#### Focus of Attention Questionnaire (FAQ; Woody, 1996)

This 10-item questionnaire asks about an individual’s focus of attention in social situations. Participants were asked to think about social situations in the past week and consider what they were focusing on. Participants responded to five items about the self (such as “I was focusing on my level of anxiety”) and five items about things outside the self (such as “I was focusing on the other person’s appearance or dress”) on a 5-point scale from “Not at all” to “Totally”. For this study, we only analysed responses to the FAQ-self subscale. This measure was not developed for use in therapy according to the Clark and Wells model of social anxiety, but has been used in studies validating the model (e.g., Hodson et al., 2008).

### Data Analysis

Analysis was completed using R (R Core Team, 2022), assisted by several R packages: psych (Revelle, 2022), mice (van Buuren & Groothuis-Oudshoorn, 2011) and lavaan (Rosseel, 2012).

We assessed the psychometric properties of social anxiety measures used in the study, as these were key to our analysis and several had not previously been used with autistic people. We assessed internal consistency by calculating Cronbach’s alpha (and 95% CI) and item-total correlations (totals not including the item) in both Group 1 (individuals diagnosed with autism) and Group 2 (“neurotypical” individuals). We assessed convergent validity by calculating correlations with a measure of anxiety, the ASA-A. We take the ASA-A as a valid measure of anxiety in autistic people, given that it was validated with and for this group. We assessed discriminant validity by calculating correlations between LSAS-SR and a depression measure, the HADS-D, expecting correlations to be lower with depression than anxiety.

#### Hypothesis Testing: Do Cognitive-Behavioural Features of The Clark and Wells Model Account for Social Fears in Autistic People?

We tested this hypothesis using a hierarchical multiple regression with LSAS-SR scores as criterion variable, and included individuals diagnosed with autism and the neurotypical control group (i.e., Groups 1 and 2) in the analysis. Multiple imputation was used to deal with a small amount of missing data on the LSAS-SR (0.4% of data) and FAQ-self (2.7% of data). Regression diagnostics were run for the analysis to check its robustness.

In the first stage of the regression, predictors were SCQ, SBQ and FAQ-self scores. In the second stage, presence of an autism diagnosis (dichotomously-coded) was added as a predictor. In the third stage, interaction terms between autism diagnosis and the other three variables were added to the regression. We tested whether the two later steps explained significantly more variance than the previous step using an *F*-test, and report adjusted *R*^*2*^ values for each step. If presence of an autism diagnosis is a significant predictor, this suggests that autistic people experience greater social fears than expected based on variables of the Clark and Wells model. Likewise, if interactions are significant, this suggests that the Clark and Wells variables are differentially linked to social anxiety in autism.

#### Do Cognitive-Behavioural Features of The Clark and Wells Model Explain the Link Between Autistic Traits and Social Fears?

As Analysis (2) suggested some differences between autistic and non-autistic people, we looked again at the link between autism and social fears in more exploratory analysis, this time defining autism in terms of autistic traits across the sample. Effectively, this allowed us to carry out analysis across the full sample including Groups 3 and 4 as well. We examined the extent to which Clark and Wells variables (SCQ, SBQ and FAQ-self) explained the link between autistic traits (measured by the AQ-10) and LSAS-SR scores. We expected AQ-10 scores only to be partially explain, as autistic traits may exert an influence on social fears in ways not accounted for by the Clark and Wells model. A path analysis using maximum likelihood estimation (with robust standard errors and a Satorra-Bentler scaled test statistic) was used to test this hypothesis. It should be noted that the cross-sectional nature of our data means that this analysis cannot be interpreted as a formal mediation analysis. We cannot say that autistic traits “cause” development of increased social fears via the Clark and Wells variables, as this would require the different constructs to be measured at different points in time.

For the path analysis, indirect paths were set between the AQ-10 and LSAS-SR via the SCQ, SBQ and FAQ-self variables. The SCQ, SBQ and FAQ-self were allowed to co-vary. A direct path was also included between AQ-10 and LSAS-SR. We then constrained the model by setting the direct path between the AQ-10 and LSAS-SR to zero. Model fit of this more constrained model was compared to the full model using a chi-square test. A significant chi-square test indicated only partial mediation, and we computed percent mediation (with 95% CI). Fit statistics (CFI and RMSEA with 90% CI) were also computed.

## Results

See Table 3 for psychometric properties of the social anxiety measures in autistic and non-autistic people.

**Table 3:** Psychometric Properties of Social Anxiety Measures

Measure	Group 1		Group 2	
	Cronbach’s alpha [95% CI]	Median item-total correlation [min, max]	Cronbach’s alpha [95% CI]	Median item-total correlation [min, max]
LSAS-SR	0.94 [0.93, 0.95]	0.63 [0.49, 0.71]	0.95 [0.94, 0.97]	0.67 [0.39, 0.83]
SCQ	0.94 [0.93, 0.95]	0.62 [0.48, 0.74]	0.95 [0.94, 0.97]	0.69 [0.53, 0.78]
SBQ (original)	0.88 [0.85, 0.90]	0.48 [-0.02, 0.63]	0.91 [0.88, 0.94]	0.57 [-0.19, 0.76]
SBQ (revised)	0.90 [0.88, 0.92]	0.50 [0.29, 0.65]	0.93 [0.91, 0.95]	0.61 [0.31, 0.78]
FAQ-self	0.76 [0.70, 0.81]	0.53 [0.44, 0.60]	0.84 [0.77, 0.89]	0.66 [0.49, 0.76]

*Note. LSAS-SR = Liebowitz Social Anxiety Scale-Self Report; SCQ = Social Cognitions Questionnaire; SBQ = Social Behaviours Questionnaire; FAQ = Focus of Attention Questionnaire*

Cronbach’s alpha was a little lower for FAQ-self, although this is to be expected given that the measure is only five items. As Cronbach’s alpha for FAQ-self was over 0.70 in both groups, this was judged adequate. On the SBQ, there were four items with negative or minimal correlations with total scores (-0.19 ≤ r ≤ .12) in both groups, suggesting they measured different constructs. These items were excluded when computing SBQ totals. Overall, all variables showed good internal reliability. We assessed convergent and discriminant validity of the social anxiety measures with another anxiety measure (ASA-A) and a depression measure (HADS-D). Correlations with the ASA-A were considerably higher than with the HADS-D for all measures, as shown in Table 4. In addition, we checked whether correlations with the ASA-A differed by gender in the autistic group. There were no significant differences (median difference in *r* = .04). This suggests that a similar pattern of relationships exists between variables relevant to social anxiety in autistic men and women. In summary, the social anxiety measures all showed good internal consistency in both groups, and appeared to be valid measures of anxiety across groups.

**Table 4:** Convergent and Discriminant Validity of Social Anxiety Measures

Measure	Group 1		Group 2	
	Correlation with ASA-A	Correlation with HADS-D	Correlation with ASA-A	Correlation with HADS-D
LSAS-SR	0.56	0.32	0.67	0.27
SCQ	0.68	0.26	0.79	0.47
SBQ	0.64	0.21	0.81	0.34
FAQ-self	0.64	0.22	0.85	0.29

*Note. LSAS-SR = Liebowitz Social Anxiety Scale-Self Report; SCQ = Social Cognitions Questionnaire; SBQ = Social Behaviours Questionnaire; FAQ = Focus of Attention Questionnaire*

See Table 5 for descriptive statistics for all variables by the four groups. Skew and kurtosis did not exceed 1 for any variable, so we take distributions as being approximately normal. There were no differences in age, gender, race/ethnicity or level of education when comparing individuals with an autism diagnosis and the nonautistic group, all *p*s > 0.05.

**Table 5:** Descriptive Statistics by Group

Variable	Group 1, Clinical diagnosis of autism (N = 192)		Group 2, Non-autistic group (N = 69)		Standardized difference between Groups 1 and 2 ^a^	Group 3, Self-identifying as autistic (N = 51)		Group 4, Broad autism phenotype (N = 24)	
	Mean	SD	Mean	SD		Mean	SD	Mean	SD
AQ-10	7.89	1.79	2.52	1.54	3.11	7.71	1.81	6.71	0.86
ASA-A	33.2	11.62	20.61	11.67	1.08	30.54	11.25	27.75	13.60
HADS-D	7.48	4.69	5.58	3.96	0.42	7.33	4.26	8.96	5.62
GAFS-8	28.98	7.00	19.88	7.45	1.28	29.84	5.45	26.79	8.51
CAT-Q	119.26	24.14	92.29	25.23	1.10	119.86	19.43	113.96	22.01
LSAS-SR ^b^	46.17	15.19	30.47	16.49	1.01	45.00	14.36	42.45	16.96
SCQ	69.37	19.47	53.59	20.06	0.80	66.94	20.38	66.62	22.19
SBQ ^c^	39.12	12.75	27.37	13.66	0.90	39.90	11.20	34.29	15.39
FAQ-self ^d^	14.28	4.31	11.68	4.52	0.60	14.10	4.92	13.79	4.20

*Note. AQ-10 = Autism Spectrum Quotient – 10 item version; GAFS-8 = General Alexithymia Factor Score; ASA-A = Anxiety Scale for Autism-Adults; HADS = Hospital Anxiety and Depression Scale; CAT-Q = Camouflaging Autistic Traits-Questionnaire; VEQ = Vulnerability Experiences Quotient; LSAS-SR = Liebowitz Social Anxiety Scale-Self Report; SCQ = Social Cognitions Questionnaire; SBQ = Social Behaviours Questionnaire; FAQ = Focus of Attention Questionnaire*

^a^ Cohen’s *d*, all *p*s < 0.05

^b^ Group 2 (N = 68)

^c^ Group 3 (N = 49)

^d^ Group 1 (N = 186), Group 2 (N = 68)

Prior to hypothesis-testing, we assessed whether there were differences on the LSAS-SR (as this was our key variable) by demographic factors. Age was not associated with LSAS-SR scores, *r* = .01, *p* = .884. The three-level variable for gender (male, female, non-binary) also did not predict LSAS-SR scores, *F* (2, 343) = 0.62, *p* = .537. These conclusions remained unchanged when these analyses were also run in the individual groups. As age and gender were not related to social fears in our sample, these variables were not controlled for in our main analyses.

### Hypothesis Testing: Do Cognitive-Behavioural Features of the Clark and Wells Model Account for Social Fears in Autistic People?

A hierarchical multiple regression tested this hypothesis. The first stage of the regression was significant, *F*(3, 257) = 84.69, *p* < .001. This showed that cognitive-behavioural features of the Clark and Wells model accounted for 49.1% of the variance in social fears across the sample. In the second stage of the regression, presence of an autism diagnosis was added to the regression as another predictor, significantly improving the model, *F*(1, 256) = 12.21, *p* < .001. Addition of this predictor explained a further 2.2% of variance. Crucially, autism diagnosis emerged as a significant predictor in this stage of the regression, *β* = -0.36, *p* < .001. This indicated that the autistic group had greater social fears than the non-autistic group, with a medium effect size, even controlling for features of the Clark and Wells model, suggesting that this model does not fully account for social anxiety in autistic people. In the third stage, we added interaction terms between autism diagnosis and each of the Clark and Wells variables to the regression. This also significantly improved the model, *F* (3, 253) = 3.10, *p* = .027. Addition of these predictors explained a further 1.1% of variance. In this analysis, autism diagnosis remained a significant predictor, *β* = -0.26, *p* = .038, and the interaction between autism diagnosis and safety behaviours (as measured by the SBQ) was also significant, *β* = 0.49, *p* = .007. This latter result indicated that there was a significantly lower relationship between safety behaviours and social fears in autistic people compared to non-autistic people when controlling for other factors. Inspection of the residuals indicated that their distribution was approximately normal and homoscedastic. Table 6 shows significance of the individual predictors in each stage of the regression.

**Table 6:** Results of Multiple Regression predicting social fears on the LSAS-SR.

Predictor	Unstandardized *β*	*SE*	Standardized *β*	*p*-value
Stage 1	* F* (3, 257) = 85.08, *p* < .001, Adjusted *R*^2^ = 0.49			
**SCQ**	**0.27**	**0.06**	**0.33**	**< 0.001**
**SBQ**	**0.55**	**0.09**	**0.45**	**< 0.001**
FAQ-self	-0.21	0.25	-0.06	0.399
Stage 2	* F* (4, 256) = 69.34, *p* < .001, Adjusted *R*^2^ = 0.51			
**SCQ**	**0.25**	**0.06**	**0.31**	**< 0.001**
**SBQ**	**0.49**	**0.09**	**0.40**	**< 0.001**
FAQ-self	-0.17	0.25	-0.04	0.495
**Group (1, 2)**	**-6.12**	**1.80**	**-0.36**	**< 0.001**
Stage 3	* F* (7, 253) = 41.96, *p* < .001, Adjusted *R*^2^ = 0.52			
**SCQ**	**0.29**	**0.07**	**0.36**	**< 0.001**
**SBQ**	**0.35**	**0.11**	**0.28**	**0.001**
FAQ-self	-0.13	0.27	− 0.03	0.638
**Group (1, 2)**	**-11.35**	**5.43**	**-0.26**	**0.038**
SCQ : Group (1, 2)	-0.17	0.15	-0.21	0.255
**SBQ : Group (1, 2)**	**0.59**	**0.22**	**0.49**	**0.007**
FAQ-self : Group (1, 2)	-0.24	0.59	-0.06	0.687

*Note. LSAS-SR = Liebowitz Social Anxiety Scale-Self Report; SCQ = Social Cognitions Questionnaire; SBQ = Social Behaviours Questionnaire; FAQ = Focus of Attention Questionnaire*

### Do Cognitive-Behavioural Features of the Clark and Wells Model Explain the Link Between Autistic Traits and Social Fears?

**Fig. 1:**
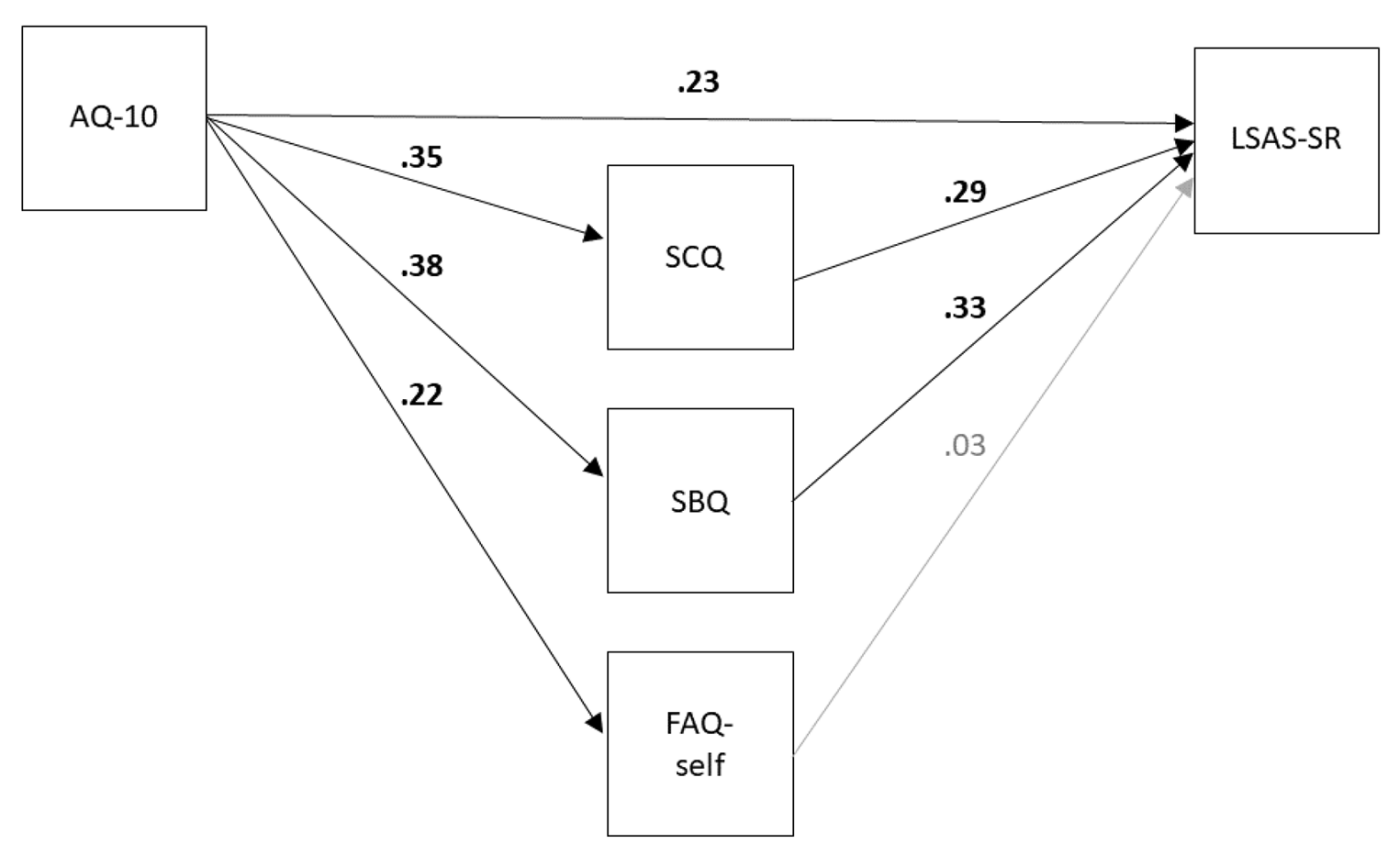
Path Diagram of a Structural Equation Model Showing the Relationship between Autistic Traits and Social Fears. *Note. AQ-10 = Autism Spectrum Quotient – 10 item version; LSAS-SR = Liebowitz Social Anxiety Scale-Self Report; SCQ = Social Cognitions Questionnaire; SBQ = Social Behaviours Questionnaire; FAQ = Focus of Attention Questionnaire*

Figure 1 shows the path analysis fitted to the data set. We ran this path analysis in a more constrained version to test for full mediation by fixing the direct path between the AQ-10 and LSAS-SR to zero. This model represented a significant reduction in fit compared to the unconstrained model, Χ^2^ (1) = 27.20, *p* < .001, demonstrating that the effect of autistic traits on social fears was not fully explained by other variables in the model. Fit statistics were as follows for this constrained model: Robust CFI = 0.97, Robust RMSEA = 0.30 [0.21, 0.40]. Including a mediation term in the path analysis indicated that other variables explained 50% of the effect of AQ-10 scores on LSAS-SR, 95% CI [36%, 64%].

As the variables included in the model explained only half the relationship between autistic traits and social fears, we were curious if other theoretically linked variables explained any further proportion of the relationship. We therefore added the VEQ-subset, CAT-Q, GAFS-8 and ASA-A anxious arousal and uncertainty subscales one by one as mediators between the AQ-10 and LSAS-SR. When controlling for the other variables in the model, only the path between the ASA-A uncertainty scale and LSAS-SR was significant, *β* = 0.19, *p* = .005. With this additional mediator, 66% of the effect of the AQ-10 on LSAS-SR was explained by indirect effects, 95% CI [47%, 86%].

## Discussion

Our analysis presents a mixed picture of social anxiety in autistic people, with evidence of similarity and difference compared to neurotypical people. On the one hand, the Clark and Wells model seems highly relevant to autistic people. In the multiple regression, Clark and Wells variables explained almost half the variance in social fears, with the addition of autism related variables only explaining a small amount of additional variance. This would suggest substantial similarity between autistic and nonautistic people in terms of their experience of social anxiety, with only minimal differences. However, the significance of individual predictors in this multiple regression tells a different story, as (1) autism diagnosis and (2) the interaction between autism diagnosis and safety behaviours were both significant predictors; this tells us that we need to know whether a person is autistic or not to make the best prediction of their level of social fears. In addition, in the structural equation model, autistic traits were a significant predictor of social fears, even accounting for the cognitive-behavioural features of the Clark and Wells model.

Both these analyses suggest that autism influences the presentation of social anxiety in ways that are at least somewhat distinct from the Clark and Wells variables, which supports previous research suggesting there are autism-specific anxiety presentations (Kerns et al., 2014). This may be partly due to cognitive differences in autism which may influence anxiety sensitivity and the ways people respond to emotions in ways specific to autism (South & Rodgers, 2017; Stark et al., 2021). However, it is also likely that systemic factors of living in a world that does not always understand or respond well to autism contribute to anxiety specifically in autistic people (Botha & Frost, 2020), especially anxiety that is social and relational in nature. In this respect, we found that people with greater autistic traits were more likely to report negative early social experiences, which agrees with previous research into incidence of childhood victimisation in autistic people (Sreckovic et al., 2014). Therefore, people are likely to learn damaging lessons about themselves and other people as they grow up, which may have a later impact on social fears.

### Similarities Between Autistic and Non-autistic People

Autistic people resonated with the key aspects of the Clark and Wells model, reporting a high frequency of negative thoughts in social situations, as well as high use of safety behaviours and self-focused attention in social situations. It is worth underscoring that autistic people in our sample very commonly endorsed negative thoughts on the SCQ, many of which relate to negative evaluation by others (e.g., being seen as weird or stupid). These thoughts were highly predictive of social fears in both autistic and non-autistic people. This is an important point, as previous research has suggested that autistic people might experience social distress in the absence of fears of negative evaluation (e.g. Kerns et al., 2014). However, our analysis suggests that such thoughts are in fact central to social anxiety in autism, which is comparable with the neurotypical population.

Interestingly, self-focused attention did not uniquely predict social fears in either autistic or non-autistic people, despite this being suggested in the Clark and Wells model. In other studies, self-focused attention has been found to contribute unique variance in predicting social anxiety (Hodson et al., 2008) and to mediate improvements in anxiety in therapy (Mörtberg et al., 2015). By contrast, this study found that self-focused attention was a less central variable. It is difficult to interpret this finding, as it was the case in both autistic and non-autistic people. Perhaps, the study was underpowered to detect a specific effect of self-focused attention if the effect is relatively small (and especially if it is smaller in neurodivergent people).

### Differences Between Autistic and Non-autistic People

There are several clues in our analysis indicating that the Clark and Wells model does not tell the whole story of social anxiety in autism. Firstly, the presence of a diagnosis of autism was a significant predictor of social fears even when controlling for features of the Clark and Wells model in the multiple regression. This suggests that factors not currently captured by the model are important in understanding social anxiety specifically in autistic people, who experience greater social fears than predicted by the model. In addition, factors included in the Clark and Wells model may differ in importance in autism. In our sample, there was a weaker relationship between safety behaviours (measured by the SBQ) and social fears in autistic compared to non-autistic people. The reason for this is not clear but it is possible that some behaviours on the SBQ that may be motivated by anxiety in non-autistic people (e.g., staying on the edge in groups) may have other functions in autistic people, for example dealing with sensory overwhelm or reflecting different social/communication preferences. There may also be a similarity between safety behaviours and camouflaging behaviours, which may be necessary adaptations to difficult social environments, with a range of purposes and consequences for autistic people, and not just reflect social anxiety (Cage & Troxell-Whitman, 2019). In addition, anxiety may be generally elevated in autism for other reasons, so safety behaviours may not be such a key maintaining factor. This has important clinical implications, as one of the most important ingredients of therapy according to the Clark and Wells model involves behavioural experiments where a person tries using fewer safety behaviours to test out negative expectations about social situations (Schreiber et al., 2015). However, if safety behaviours are less crucial in autism, this focus on safety behaviours may not be appropriate.

As we have noted, the Clark and Wells model did not entirely fit for people with a clinical diagnosis of autism, and in addition, it was only partly applicable when defining autism in terms of traits varying across the population. In this analysis, we found that the Clark and Wells variables accounted for just half the link between autistic traits and social fears, suggesting there must be other variables involved. In exploratory analysis, we examined whether other theoretically-relevant variables might explain part of the link between autistic traits and social fears. We found that alexithymia, camouflaging, anxious arousal and negative early social experiences did not explain additional variance, but uncertainty distress did. This latter finding agrees with previous research, which has found robust evidence for elevated intolerance of uncertainty in autism and a strong link with anxiety (Jenkinson et al., 2020). Intolerance of uncertainty relates to various aspects of the autism phenotype, including difficulties with pragmatics and social communication (Wilson & Bishop, 2022), and sensory sensitivities and preference for sameness (Hwang et al., 2020). Given that these features may be issues for autistic people in social environments, it makes sense that uncertainty distress is another factor in social fears. However, uncertainty distress only accounted for a small amount of additional variance, and there will be other relevant factors too. In a companion paper (Wilson & Gullon-Scott, 2023), we offer some qualitative analysis of comments made by participants on free response questions in the survey, which gives some possible hints of important other factors.

Based on the quantitative analysis reported here, it is difficult to recommend specific adaptations to therapy for social anxiety for autistic people, as this paper has purely focused on testing for equivalence between autistic and non-autistic people in experiences of social anxiety. By contrast, our companion paper analysing the qualitative data is likely to have more practical suggestions for practitioners working with autistic people. Nonetheless, there are a couple of key take-home messages for therapists from the analyses reported here:

- Do not assume that autistic people will necessarily benefit from therapy for social anxiety according to the Clark and Wells model. Help people understand the model, so that you can make a decision together about whether it is likely to help them, and evaluate as you go. Appreciate that there are likely to be autism-specific factors that may be important, as well as factors in the existing model that may be less relevant or indeed inappropriate. (For instance, the analysis reported here suggests that distress relating to uncertainty has a role in social anxiety for autistic people, and that safety behaviours may not be equivalent between autistic and non-autistic people.)
- Do not assume that autistic people will *not *benefit from therapy for social anxiety according to the Clark and Wells model. There is good evidence in this paper that the factors included in the model are relevant to autistic people, as they reported elevated levels for all factors (socially-relevant negative thoughts, safety behaviours, and self-focused attention). If these can be effectively targeted in therapy, autistic people may experience a reduction in social fears.

#### Limitations

This is the first piece of research to directly address whether a specific model of social anxiety applies to autistic people. A significant proportion of autistic people experience social anxiety (Spain et al., 2018), so this is an important area of research, and is one prioritised by autistic people themselves (James Lind Alliance & Autistica Research Priority Setting Partnership, 2015). Our sample was large, and included autistic people who were clinically or self-diagnosed. Some groups were under-represented in our research: men, people who are not White, and people with less educational advantage. In terms of gender, we found no differences in the correlations between different elements of social anxiety in autistic men and women, suggesting that the over-representation of women in the sample may not be an issue. By contrast, the lack of individuals with a co-occurring learning disability *is* likely to be an issue for generalising results to this group, as we had very few (if any) people with a learning disability take part. It is possible that the length and literacy demands of our survey might have limited the ability for some people to engage. Therefore, it will be important that future research particularly reaches out to this group of people. We should also bear in mind that there may be limitations in the measures used for the different constructs of the Clark and Wells model in this study, as they were not developed with autistic people, so it is possible that there were some thoughts/behaviours specific to autism not included in them. However, overall, the psychometric properties of the measures were very strong, so this is probably not too problematic. In addition, we only measured the different constructs at one point in time, so we are unable to make any conclusions regarding causality or precedence. For instance, the structural equation model should not be taken as evidence that autistic traits “cause” the development of social fears.

## Conclusion

This study explored whether one of the leading models of social anxiety in non-autistic people also applies to autistic people. We found that the Clark and Wells model is likely to be relevant to social anxiety experienced by autistic people, but it is not the whole story. We found that autistic people experience greater social fears even when controlling for their experience of variables of the Clark and Wells model (negative social thoughts, safety behaviours and self-focused attention). This suggests that other variables not accounted for by the Clark and Wells model are also important in explaining why being autistic increases risk for social fears. It was a very similar message when we looked at the link between traits (rather than a diagnosis) of autism and social fears, and found that the Clark and Wells variables only partly explained the link. Therefore, we suggest that therapists bear in mind that the Clark and Wells model may not provide an exact fit for autistic people. Future research will need to further unpack the autism-specific mechanisms that account for social anxiety, while applying this understanding to adapt therapy in the most appropriate ways for autistic people.
